# Depression Following Thrombotic Cardiovascular Events in Elderly Medicare Beneficiaries: Risk of Morbidity and Mortality

**DOI:** 10.4061/2009/194528

**Published:** 2009-12-22

**Authors:** Christopher M. Blanchette, Linda Simoni-Wastila, Fadia T. Shaya, Denise Orwig, Jason Noel, Bruce Stuart

**Affiliations:** ^1^Division of Clinical and Outcomes Research, Lovelace Respiratory Research Institute, Albuquerque, NM 87108, USA; ^2^Division of Pharmaceutical Outcomes and Policy, University of North Carolina School of Pharmacy, Chapel Hill, NC 27599, USA; ^3^Department of Pharmaceutical Health Services Research, School of Pharmacy, University of Maryland, Baltimore, MD 21201, USA; ^4^Department of Epidemiology and Preventive Medicine, School of Medicine, University of Maryland, Baltimore, MD 21201, USA; ^5^Department of Pharmacy Practice, School of Pharmacy, University of Maryland, Baltimore, MD 21201, USA

## Abstract

*Purpose*. Depression and antidepressant use may independently increase the risk of acute myocardial infarction and mortality in adults. However, no studies have looked at the effect of depression on a broader thrombotic event outcome, assessed antidepressant use, or evaluated elderly adults. *Methods*. A cohort of 7,051 community-dwelling elderly beneficiaries who experienced a thrombotic cardiovascular event (TCE) were pooled from the 1997 to 2002 Medicare Current Beneficiary Survey and followed for 12 months. Baseline characteristics, antidepressant utilization, and death were ascertained from the survey, while indexed TCE, recurrent TCE, and depression (within 6 months of indexed TCE) were taken from ICD-9 codes on Medicare claims. Time to death and first recurrent TCE were assessed using descriptive and multivariate statistics. *Results*. Of the elders with a depression claim, 71.6% had a recurrent TCE and 4.7% died within 12 months of their indexed TCE, compared to 67.6% and 3.9% of those elders without a depression claim. Of the antidepressant users, 72.6% experienced a recurrent TCE and 3.9% died, compared to 73.7% and 4.6% in the subset of selective serotonin reuptake inhibitor (SSRI) users. Depression was associated with a shorter time to death (*P* = .008) in the unadjusted analysis. However, all adjusted comparisons revealed no effect by depression, antidepressant use, or SSRI use. *Conclusions*. Depression was not associated with time to death or recurrent TCEs in this study. Antidepressant use, including measures of any antidepressant use and SSRI use, was not associated with shorter time to death or recurrent TCE.

## 1. Introduction

Depression affects one out of every three elders [1, 2] and is associated with substantial societal and medical costs [2–4]. Depressions leads to reductions in functional status and productivity [5] and increases the risk of comorbidity [5] and cardiac mortality [6]. Thrombotic cardiovascular events (TCE) may play a key role in the etiology of depression and subsequent morbidity and mortality due to factors associated with the event, including decreased functional status, social isolation, comorbid chronic medical conditions, a sense of death, and physiological processes [7–9]. It is unclear whether pharmacological treatment for depression decreases the risk of future cardiac complications. 

### 1.1. Depression and Cardiac Events

Few studies have measured the effect of depression on recurrent cardiac events following a TCE. Only one study in the elderly emerged. A study of Japanese elders stratified by depression and followed for 12 months found the annualized cardiac event rate to be 7.3% higher in depressed elders compared to nondepressed elders [10]. Only one similar study was found in nonelders [11].

### 1.2. Depression and Cardiac Mortality

Depression in elders leads to higher rates of cardiac mortality compared to nondepressed elders. Although most studies do not differentiate between elders and adults, there are some studies that specifically assess cardiac mortality in elders with depression after a TCE. Bush et al. found that depressed elders with left ventricular ejection fraction <35% after an AMI had a 4-month mortality rate of 50%, compared to the 12% mortality rate of similar patients without depression [12]. Welin et al. found that elders with higher scores on the BDI (indicating more severe depression), who had experienced an AMI had a threefold risk of coronary death compared to elders with lower scores [13]. Findings were similar in the general adult population. A study of post-AMI depressed individuals were at a significantly greater risk of cardiac mortality than post-AMI nondepressed individuals at 6 months [6], 12 months [14], 18 months [11, 15] and up to 27 years after initial depression measurement [16].

Although a substantial amount of evidence has implicated depression after a TCE as a risk factor for mortality, two studies found that depression was not associated with mortality [17, 18].

### 1.3. Antidepressant Treatment and Cardiac Events

Several studies document the relationship between depression treatment and morbidity and mortality. The Sertraline Antidepressant Heart Attack Randomized Trial (SADHART) indicated that rates of cardiovascular events were lower in the sertraline (an SSRI) group than in the placebo group (14.5% and 22.4%, resp.), which suggested the possible protective effect of SSRIs against recurrent cardiovascular events, although the rates were not statistically different enough to be significant [19]. Conflicting results were found in observational studies, with some showing an increased risk of AMI associated with tricyclic antidepressant use [20] or SSRI antidepressant use within the first 7 days [21] or if an SSRI is stopped within the month prior to the AMI [22], and others demonstrating the protective nature of SSRI antidepressants against AMI [22–24]. Still others have found no effect on AMI by tricyclic antidepressant use [24, 25], SSRI antidepressant use [25], or other antidepressant use [25]. The most important factor missing from all of these studies is the inclusion of depression in the analyses. The biologically based theories also suggest that a broader class of thrombotic events may be related to depression and not just AMI, as previous studies assess [26].

The purpose of this study was to examine whether evidence of depression after a TCE places elders at a greater risk of subsequent TCEs or mortality. This study also measures the effect antidepressant treatment (and specifically treatment with SSRIs) has on these outcomes. The analyses are conducted in a high risk population of elderly Medicare beneficiaries measuring outcomes within the first twelve months following an indexed TCE. The primary research hypothesis is that a claim for depression is associated with an increased risk of recurrent TCEs and mortality. The secondary research hypothesis is that both antidepressant and SSRI treatment for depression will also increase the risk of recurrent TCEs and mortality.

## 2. Materials and Methods

### 2.1. Data

The Medicare Current Beneficiary Survey (MCBS) is one of the richest databases available to study disease and prescription drug use in the elderly. The MCBS is a tri-annually administered survey linked to Medicare Part A and B claims. It is designed and maintained by the Centers for Medicare and Medicaid Services (CMS), the federal agency responsible for the payment of services for all Medicare and Medicaid beneficiaries. The design of the MCBS is a longitudinal rotating panel, which includes approximately 4000 long-term care (LTC) and community-dwelling beneficiaries followed for three years. Each year of the survey consists of three panels or approximately 12,000 Medicare beneficiaries. The survey contains demographic characteristics, medical history, insurance coverage, healthcare services use and expenditures, and death. The MCBS is also one of the only available sources of prescription drug use by Medicare beneficiaries. The linkable claims files contain data on diagnosis, utilization, and payment for inpatient and outpatient Medicare-covered services. An attractive feature of the MCBS is the relatively low loss to follow-up rate, only (8% to 10% annually) [27].

### 2.2. Sample Selection and Study Design

The design of the study was a historical pooled cohort of 7,051 Medicare beneficiaries aged 65 years of age and older residing in the community who experienced a TCE according to the definition of a thrombotic event set forth by Shaya et al. [28] (AMI, ischemic heart disease, acute pulmonary heart disease, acute cerebrovascular diseases, or pulmonary and venous embolisms). The cohort was pooled from elders residing in the 1997 to 2002 MCBS. Elders were excluded from the cohort if they: (1) were enrolled in a Medicare HMO (due to nonavailable claims because of third-party handling); (2) failed to complete one of the three surveys administered annually within a two year period (except when due to death) in order to accurately observe antidepressant utilization; or (3) resided in a long-term care facility during the baseline year. Individuals in long-term care facilities lack prescription drug utilization data in the MCBS, and in addition, the etiology of their depression may be very different than seen with elders residing in the community. However, if the elder transitioned to a nursing facility during the follow-up period, the elder was included in the cohort provided that all other inclusion criteria were met.

### 2.3. Variable Measurement

A monthly analytic file (numbering 1 to 72 months across the study period, January 1997 through December 2002) was created in which elders were noted as having had a TCE or depression in the month corresponding to the date of service indicated on the claim from one of three sources: inpatient, outpatient, or physician office visits. The *International Classification of Diseases, Ninth Revision*, (ICD-9) is the numerical system used to code any written description of a diagnosis or medical condition. For this study, the ICD-9 diagnostic codes listed on the claims were used to identify depression and TCEs. TCEs were identified by any ICD-9 code with the first three digits of 410, 411, 413, 414, 415, 433–438, 452, or 453. This criteria is similar to that set forth by Shaya et al. [28], which adds pulmonary and venous embolism or thrombosis (452 and 453) to the Antiplatelet Trialist Collaboration (APTC) combined endpoint [29] to ensure that all TCEs were captured. Code 798 (included in the APTC endpoint) or all-cause death was omitted from the definition in order to assess outcomes after the indexed event. Depression was identified through at least one ICD-9 diagnostic code of 296.2, 296.3, 296.5, 296.6, 298.0, 300.4, 308.0, 309.0, 309.1, 309.4, or 311 on any claim submitted for reimbursement. Elders were identified as having depression if the month of the recorded depression (1 to 72) was within six months after the month of a recorded TCE.

Recurrent TCEs were assessed using the same criteria set forth for the indexed TCE. Mortality is captured in the MCBS as well as the date and source of verification. Dates of service utilization were then used to assign month indicators (0–72 over the entire observation period) corresponding to the event and used to measure time to event.

Baseline characteristics were obtained from the elder's survey corresponding to the year in which the indexed TCE was identified. These variables included age, race, sex, income, smoking history and status, height and weight, source of first Medicare entitlement, household living situation, and history of thrombotic event. All baseline characteristics were transformed into categorical data. Income was transformed based on Federal Poverty Level (FPL) criteria through weighting reported income in the survey by twenty percent due to underreporting [30], adjusting income to 2001 dollars through the use of the consumer price index [31], and then creating a binary variable cut at $8,590 according to the 2001 FPL guidelines [32]. Body mass index (BMI) was calculated using the elders reported height and weight and then categorized into the following categories indicative of frail (<25), average (25–30), and obese (>30) elders. Isolation is known to be a risk-factor for depression so elders were identified as living alone or not. Due to data limitations, it was not possible to determine if the indexed TCE was an elder's first, a survey question that asked “Have you ever been told you had a myocardial infarction/heart attack?” was assessed as a proxy for a history of a thrombotic event.

Prescription drug use also was assessed from the survey due to the lack of Medicare coverage for prescription drugs during the study period. The survey included a rigorous process of data collection that included having beneficiaries save all prescription drug packages for the interviewer to record. The beneficiaries were questioned about prescription drug use regardless of the packaging in order to minimize omitted drug use. Prescription drugs were then itemized per patient by year and listed according to the prescription, resulting in a list of drug events. Antidepressants were selected from the list of prescription drug events for elders in the year corresponding to their depression claim. In order to assess the effect of antidepressant use on depression and maintain temporal relationships between depression and antidepressant use, (since antidepressant use is annually measured in the survey and depression is measured within the first six months following a TCE) antidepressant use was not assessed for those elders without a depression claim in the main analyses. However, sensitivity analyses were conducted to address an independent effect.

Elders were categorized into the following categories: (1) “SSRI users” if they had a depression claim and at least one of the following drug events including citalopram, fluvoxamine, escitalopram, fluoxetine, paroxetine, and sertraline; or (2) “other non-SSRI antidepressant users” if they had a depression claim, were not previously identified as a SSRI users, and had a drug event for any other antidepressant including amitriptyline, amoxapine, clomipramine, desipramine, doxepin, imipramine, nortriptyline, protriptyline, trimipramine, buproprion, maprotiline, mirtazapine, nefazodone, phenelzine, tranylcypromine, trazodone, or venlafaxine in the corresponding year of the depression claim; and (3) “antidepressant user” if the elder had any of the above antidepressant events listed above in the year of the depression claim.

### 2.4. Analyses

Analyses included descriptive statistics, bivariate comparisons (Chi-square tests and Kaplan-Meier survival plots), and multivariate statistics (Cox-proportional hazard regression models). All statistical analyses were completed using SAS version 9.1 (Cary, NC). Alpha was set at 0.05 a priori for tests of significance.

Descriptive statistics of baseline factors, recurrent events, and mortality were conducted to display counts and percentages for corresponding groups and measures of central tendency including mean, median, and standard deviation for those elders who experienced the corresponding event. Kaplan Meier survival plots were used to measure the time to first recurrent event as well as time to death by study groups including (1) depressed and nondepressed elders; (2) nondepressed elders, antidepressant users, and depressed nonantidepressant users; and (3) nondepressed elders, other antidepressant users, and SSRI users. Chi-square tests were similarly calculated for each group.

Cox-proportional hazard models were used to measure the time to first recurrent TCE and time to death while controlling for baseline characteristics. Three models were run for each event while controlling for previous healthcare spending, age, race, gender, smoking status and history, poverty status, BMI, and baseline year. The primary variable of interest in model 1 was a claim for depression, model 2 was both a claim for depression and antidepressant use by the depressed elders, and model 3 was a claim for depression and SSRI use by the depressed elders.

Sensitivity analyses were conducted to measure the independent effect of antidepressant use on the outcomes and a sub-analysis was conducted to observe the effect of antidepressant use by reducing or eliminating the threat of misclassification bias. The sensitivity analyses included any antidepressant use/SSRI use in the Cox-proportional hazard models (similar to model 2 and 3) not just associated with a depression claim. In the sub-analyses, both model 2 and 3 were repeated with the subset of patients who had a claim for depression in an attempt to reduce or remove potential misclassification bias introduced by the absence of a depression claim for truly depressed elders.

The authors had full access to the data and take responsibility for its integrity. All authors have read and agreed to the manuscript as written.

## 3. Results

### 3.1. Baseline Characteristics

Table 1 presents baseline characteristics of the cohort. Elders were similarly distributed across age ranges, except for those aged 80 and older (41.2%). The racial/ethnic composition of the sample was as expected, with Caucasian being the most prevalent (87.6%), followed by African American (8.5%), and then other races (3.6%). Women made up slightly less of the sample (44.9%) than men (55.5%). Over half of the sample had a history of smoking (51.6%) while very few reported currently smoking (10.4%). Approximately one-third of the elders in the cohort (30.8%) claimed to have had an AMI in the past.

### 3.2. Descriptive Morbidity and Mortality Rates

Of the 6,671 elders who did not have a depression claim, 67.6% had recurrent TCEs and 3.9% died within one year of the indexed TCE. Of the 380 elders with a depression claim, 71.6% had recurrent TCEs and a slightly higher mortality rate (4.7%). Antidepressant users as a whole had a slightly higher rate of recurrent TCEs than either of the previous groups, but a lower mortality rate than the larger depressed group (3.9%) Non-SSRI antidepressant users experienced a lower rate of mortality (1.6%), which accounted for only one death. SSRI users experienced a greater rate of recurrent TCEs compared to non-SSRI users, 73.7% to 68.9%, respectively. Time to recurrent event was also slightly greater for the SSRI group compared to the non-SSRI group, with a mean time of 3.1 months compared to 2.5 months.

### 3.3. Unadjusted Analysis

**Figure 1:**
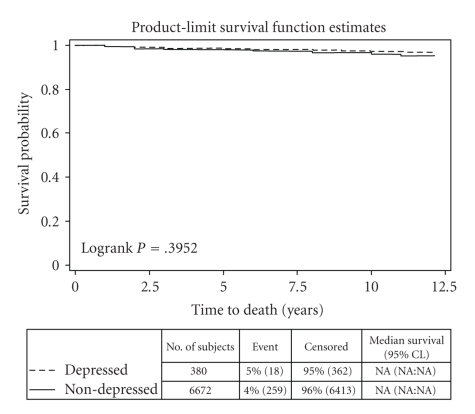
Time to death by group.

**Figure 2:**
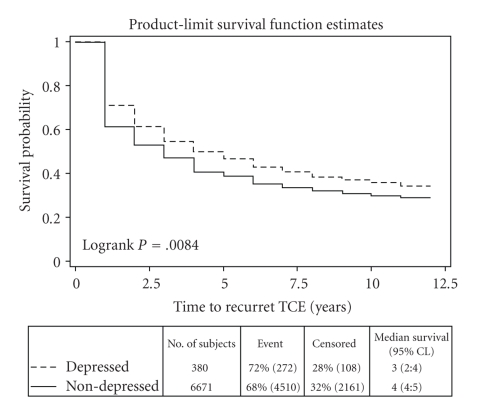
Time to recurrent TCE by group.

Kaplan Meier survival plots showed that elders with a depression claim had a recurrent TCE sooner than other elders (Logrank *P* = .008). However, there was no difference in time to death between elders with a depression claim and those without. There also was no difference in time to recurrent TCE or death between any of the other subgroups of elders with a depression claim, antidepressant user compared to nonantidepressant user or SSRI users compared with other antidepressant users. Antidepressant users and SSRI users were compared to elders without depression claims and no difference was found in time to either recurrent TCE or death. Chi-square statistics were also calculated between each group for unadjusted comparisons of counts with similar nonsignificant findings (Figures 1 and 2).

### 3.4. Multivariate Analysis

The first model measuring the association between a depression claim and time to recurrent TCE while controlling for key factors (previous healthcare spending, age, race, gender, smoking status and history, poverty status, BMI, and baseline year) revealed that the effect seen earlier in the unadjusted Kaplan Meier survival curves between the group with a depression claim and other elders was explained by the covariates in the model. As a result, the hazard rate was very close to 1.00 with an insignificant *P*-value. When adding any antidepressant use in the second model, we similarly found an insignificant effect of depression claim, as well as no effect by antidepressant use. These results were identical when antidepressant use was replaced with SSRI use.

Similar models used to assess time to death between the groups and antidepressant sub-groups found more movement among the hazard ratios but none were statistically significant. When antidepressant use was added into the model, the hazard rate for a depression claim increased to 1.52 and the hazard rate for antidepressant use showed a protective effect at 0.52; neither finding was statistically significant. Similar findings resulted when antidepressant use was replaced with SSRI use in the model. 

There were a few other factors in the models that consistently showed an effect on the outcomes. Those found to significantly decrease the time to a recurrent TCE included previous healthcare spending and being male. Factors found to significantly decrease the time to death were previous healthcare spending, being older than 80 years, and having a BMI greater than or equal to 25.

Results from the sensitivity analyses reveal similar findings to those of the other models, with the exception of any antidepressant use. It was significantly associated with an increase in time to death in the followup period (HR = 0.66, CI: 0.47, 0.94), however when antidepressant use was replaced with SSRI use, this effect became nonsignificant. Neither was significantly associated with time to recurrent TCE. The sub-analyses of elders with only a depression claim also revealed no effect on recurrent TCE or death by antidepressant use or SSRI use. 

## 4. Discussion

Neither a depression claim nor antidepressant use was found to be associated with time to recurrent TCEs or death in this sample of elderly Medicare beneficiaries. This study indicated that a claim for depression within six months after experiencing a TCE did not decrease time to another TCE or death within one year in an elderly Medicare sample. Similarly, the use of antidepressants, including this study's measures of any antidepressant use and SSRI-specific use, was not associated with shorter time to recurrent TCE or mortality within one year of the indexed TCE.

There are several key reasons why these data may differ from those of previous studies, which may include demographic and baseline characteristics of our cohort, measures of depression, length of followup time, and key limitations inherent of claims data and observational studies. 

Baseline characteristics were similar to those expected and without previous studies of the elderly population, they are difficult to compare. However, this study did find a higher percentage of older elders (>80 years) than expected. This was probably due to the inclusion of cerebrovascular disease into the definition of the TCE, which defined the cohort. Previous studies focused primarily on associations between depression or antidepressant use on either stroke or AMI and not the larger group of TCEs [12, 13]. Based on the literature [26], the link between depression and the cardiovascular system could be assumed to affect all TCEs. However, the effect may in fact depend on specific TCEs, like AMI or stroke. This study represented elderly adults, while most of the previous studies focused on the younger adult population [6, 11, 16, 18]. The only factors found to significantly decrease the time to a recurrent TCE were previous healthcare spending and being male; the factors found to significantly decrease the time to death were previous healthcare spending, being older than 80 years, and having a BMI greater than or equal to 25. Variables not included in the analysis due to data limitations, but may have been important include Killip class and Peel Index score (clinically-defined severity measures of a cardiac event) [33].

Differences in the measurement of depression may have impacted study findings not only because different populations were observed, but also an effect of cross-overs due to misclassification bias. This was evidenced by the much lower prevalence of depression in this study (5%) compared to previous studies reporting much higher rates (up to 49%) [10]. Most studies use screening instruments to assess depression rather than psychological exams; most of which have not been validated in this population [6, 11, 16, 18]. Accurate depression measurement is critical, as patients who suffer TCEs are often met with functional restrictions that may increase the likelihood of being scored as depressed on a nonsubjective measurement tool, especially if this assessment is done within hours or days of the event, as is the case in most hospital-based studies [34]. The limited time from event to assessment may bias the true estimate of the prevalence of depression since the time requirement for a depression diagnosis may not be observable without a history of at least fourteen days after the event [35]. The misclassification bias potentially introduced in this study would be towards null findings due to including undiagnosed depressed elders into the nondepressed group while maintaining a homogenous, and potentially a more severely depressed group. This misclassification bias may be one reason for the null findings in this study. In an attempt to observe misclassification bias introduced by the depression measurement, the rate of any observed antidepressant utilization in elders with no depression claim throughout the survey enrollment (15%) was compared to the rate of antidepressant utilization by elders with a depression claim found in this study (68%). Based on the literature providing details on the high use of antidepressants for off-label indications and indications other than depression [36], antidepressant use in elders without a depression claim were probably due to other illnesses.

Due to limitations of the data, this study was unable to account for undiagnosed and untreated depression. A depression wash-out period prior to the indexed TCE was not included primarily because incident depression was not of interest. However, persistency may be one measure of severity, and varying levels of depression severity may have more impact on outcomes than just evidence of depression after the TCEs. Environmental and genetic factors are associated with depression, as well as TCEs, but due to data limitations, measurements of persistency and severity, and also environment and genetics, were outside the scope of this study.

Administrative databases and observational study designs are flawed with biases such as errors on claims submitted for payment and selection bias when measuring drug exposure [37]. Although risk adjustment methods were used to control for selection bias, it is difficult to reduce bias introduced by omitted variables, such as the Killip class and Peel Index score described earlier. However, steps were taken to include as many covariates as possible. Also, it was impossible to determine the disease for which the treatment was prescribed. Assumptions are generally made in observational studies such as those in this study, where antidepressants used by elders with depression claims were most likely indicated for depression. The authors acknowledge that patients could have been treated with other drugs which may have had minor antidepressant action, but that was outside the scope of this study.

One reason for this study's nonsignificant findings could be due to lack of power, however, *a priori* power analysis did prove sufficient power. A power analysis conducted based on literature for recurrent TCEs estimated 309 nondepressed and 103 depressed patients would be required, with a detectable difference of 0.075, a standard deviation of 0.235, a power of 80%, with alpha set at 0.05 [10]. Similarly power appeared to be sufficient to assess mortality based on previous findings [6]; with power set at 80%, an estimated 81 nondepressed and 27 depressed patients were required for a detectable difference of 0.138, a standard deviation of 0.221 and alpha set at 0.05.

The structure of drug exposure in the MCBS limited the ability to evaluate temporal relationships, but this limitation will be remedied when Medicare Part D prescription drug claims are released. Also, although the MCBS includes cross-sectional weights for generalizing results to the population, the design of this study prevented the use of these adjustments and the cohort was analyzed as a sample which may or may not be representative of the greater population.

While this study assumed that antidepressant use and specifically SSRI use may have similar effects on depressed individuals suffering from various TCEs, this is a very broad assumption that may not be accurate. In order to assess whether a difference could be found with respect to specific TCEs, the authors replicated the models in a subset of the sample with AMI (*N* = 2,174) and found that although depression was associate with a greater risk of recurrent TCE (HR = 1.30, CI: 1.10, 1.53), in line with the literature, neither antidepressant use nor SSRI use were associated with either an increase or decrease in risk of recurrent TCE or death, similarly to previous models in all TCE patients.

This was the first study to assess the effect of depression on the risk of recurrent TCEs and mortality in a high-risk elderly Medicare sample during the 12 months following a TCE. This analysis provided evidence that depression, treated or untreated, was not significantly associated with time to recurrent TCE or death within the first 12 months following a TCE in this population, however in AMI only patients, depression may be associated with recurrent events. Future research should compare various methods of measuring depression, including persistency and severity, and effects on specific TCEs. Prescription drug claims from the Medicare Part D benefit would enable researchers to drill down to drug level details and provide better assessments of temporal relationships in larger study cohorts. Although this study did not find depression or antidepressant use to be associated with future events, depression screening should occur after a TCE in elderly adults and appropriate treatment provided and monitored.

## Figures and Tables

**Table 1:** Sample characteristics.

	#	%
Total elders	7,051	100.0
Age		
65–70	1,170	16.6
70–74	1,401	19.9
75–79	1,575	22.3
80+	2,905	41.2
Race		
Caucasian	6,176	87.6
African American	601	8.5
other	255	3.6
Sex		
male	3,883	55.1
female	3,168	44.9
Income^2^		
</= 100% FPL	2,969	42.1
>100% FPL	4,082	57.9
History of smoking	3,639	51.6
Current smoker	731	10.4
Body Mass Index (BMI)		
<25	3,175	45.0
25–30	2,554	36.2
>30	1,213	17.2
Entitlement		
disabled	94	1.3
age	6,957	98.7
Household		
alone	2,417	34.3
not alone	4,634	65.7
Cohort		
1997	1,623	23.0
1998	1,349	19.1
1999	1,368	19.4
2000	1,306	18.5
2001	1,406	19.9
History of acute myocardial infarction	2,174	30.8

*The sample includes community dwelling non-HMO elders who experienced a thrombotic cardiovascular event (ICD-9: 410, 411, 413, 414, 415, 433–438, 452, or 453) and had at least two years of data in the 1997 to 2002 Medicare Current Beneficiary Survey.

^†^Income adjusted to 2001 dollars and inflated by 20% for underreporting.

**Table 2:** Recurrent events and mortality.

	Nondepressed		Depressed		Antidepressant users		SSRI users		Other non-SSRI antidepressant users	
	#	%	#	%	#	%	#	%	#	%
Total elders	6,671	100.0	380	100.0	259	100.0	198	100.0	61	100.0

Recurrent TCEs	4,510	67.6	272	71.6	188	72.6	146	73.7	42	68.9
Mortality	259	3.9	18	4.7	10	3.9	9	4.6	1	1.6

Time to first event (months)	mean/median/std		mean/median/std		mean/median/std		mean/median/std		mean/median/std	

Recurrent TCEs	3.3/2.0/3.0		2.6/1.0/2.5		2.6/1.0/2.5		2.5/1.0/2.2		3.1/1.5/3.1	
Mortality	6.9/7.0/4.1		6.1/7.0/3.8		8.5/9.0/2.8		8.3/8.0/3.0		10.0/10.0/—	

*Measures of central tendency include only those patients with an event.

**Table 3:** Time to event analyses.

Event	Variable	Hazard Ratio	95% CI		*P*-value
Recurrent TCE					
	Model 1			
	Depression	1.09	0.96	1.23
	Model 2			
	Depression	1.07	0.86	1.33
	Antidepressant	1.02	0.79	1.32
	Model 3			
	Depression	1.02	0.85	1.22
	SSRI	1.13	0.89	1.43
	Model 4			
	Depression	1.30	1.10	1.53

Death				
	Model 1			
	Depression	1.06	0.65	1.72
	Model 2			
	Depression	1.73	0.85	3.51
	Antidepressant	0.52	0.21	1.33
	Model 3			
	Depression	1.27	0.65	2.47
	SSRI	0.83	0.33	2.10

*All four models controlled for previous healthcare spending, age, race, gender, smoking status and history, poverty status, BMI, and baseline year.

^†^Non-SSRI users are reference group in model 3; Nonantidepressant users are reference group in model 2.

^±^Ties were handled with the Exact method.
